# Waterborne parasites in Uganda: A survey in Queen Elizabeth Protected Area

**DOI:** 10.1002/puh2.142

**Published:** 2023-12-11

**Authors:** Celsus Sente, Howard Onyuth, Andrew Tamale, Bob Mali, Benigna Gabriela Namara, Jacob Gizamba Mugoya, Andrew Rwot Omara

**Affiliations:** ^1^ Department of Wildlife and Aquatic Animal Resources School of Veterinary Medicine and Animal Resources College of Veterinary Medicine Animal Resources and Biosecurity Makerere University Kampala Uganda; ^2^ Department of Microbiology School of Biomedical Sciences College of Health Sciences Makerere University Kampala Uganda; ^3^ Spatial Science Institute University of Southern California Los Angeles California USA

**Keywords:** parasites, Queen Elizabeth Protected Area, Uganda, waterborne

## Abstract

**Background:**

Pathogenic waterborne protozoa and helminths have the potential to cause infections in vulnerable populations such as children and immunocompromised individuals. Those residing in close proximity to wildlife‐protected areas in sub‐Saharan Africa, who are among the most economically disadvantaged, may have an increased susceptibility to these infections. This study aimed at detecting protozoan trophozoites/cysts, and helminth eggs in environmental and piped tap water (PTW) used by locals and tourists in the Queen Elizabeth Protected Area (QEPA) in western Uganda.

**Methods:**

Water samples were taken from the natural environment and domestic PTW sources. The samples were analysed for helminth eggs, free‐living amoeba (FLA) trophozoites/cysts, *Cryptosporidium* oocysts and Giardia cysts. The parasites were examined under the microscope, after which they were identified and counted. The data was subjected to univariate analysis to compare the prevalence rates across the different sample sites. The variables were summed using the mean and standard error of the mean.

**Results:**

The overall prevalence rates of the protozoan parasites, from highest to lowest, were as follows: FLA (56.6%), *Cryptosporidium* spp. (38.2%), *Giardia* spp. (36.5%), *Eimeria* spp. (20.3%) and *Paramecium* spp. (2.7%). Similarly, helminth parasite prevalence was as follows: Strongyle (38.2%), *Ascaris* spp. (33.3%), Trematodes (25%), Strongyloides (10.5%), *Toxocara* spp. (8.3%) and *Capillaria* spp. (3.2%).

**Conclusion:**

The presence of parasites in natural water sources inside QEPA presents a significant hazard for the contamination of domestic water. It is imperative to implement established procedures for enhancing water quality.

## Graphical abstract

As water has a multitude of functions, encompassing culinary, hygienic and recreational applications, studies looking at potentially pathogenic waterborne pathogens are crucial in order to prevent disease spread to rural populations and tourists. This study aimed at detecting protozoan trophozoites/cysts, and helminth eggs in environmental and piped tap water used by locals and tourists in the Queen Elizabeth Protected Area (QEPA) in western Uganda. Water samples were taken from natural (environmental) and domestic (piped tap water) sources. Identification of protozoan and helminth parasites in both natural and domestic water sources necessitates advocacy for the implementation of established procedures for enhancing water quality.

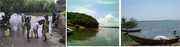

## INTRODUCTION

WASH, which stands for safe water, sanitation and hygiene, is commonly employed by non‐governmental organisations and aid agencies operating in developing countries. The purpose of the WASH programme is to emphasise the implementation of sustainable measures aimed at preventing and controlling diseases, enhancing public health, fostering community resilience and effectively addressing global emergencies and outbreaks [1]. Despite the various activities of WASH, more than 884 million people around the world still do not have access to clean water [2, 3, 4]. There are over 2.4 billion individuals worldwide who lack access to even the most fundamental sanitation services [4]. This could be attributed to the fact that many people still defecate in the open, sometimes near water sources. The lack of toilets and latrines in many areas around national parks makes the situation worse which impacts on the safety of the water supply for drinking and other uses. Water that has been contaminated with human faeces, for example as a result of rainwater wash‐off, is of particular concern, especially during rainy seasons, as it may contain organisms that are capable of causing severe illness or even death [5, 6, 7]. There are over 4 billion instances of diarrhoea reported annually around the world, 88% of which are attributed to contaminated water used for drinking [2, 4, 8–10].

Contaminated water has the ability to serve as a reservoir for several parasites, including protozoa and helminths. Among these parasites, free‐living amoeba (FLA) helminths, *Cryptosporidium* and *Giardia* are typically overlooked as waterborne parasites. For example, some types of FLA that spread through water such as *Naegleria* spp. and *Acanthamoeba* spp are responsible for primary amebic meningoencephalitis and granulomatous amoebic encephalitis, respectively [11]. Acanthamoeba is also a common cause of keratitis that often leads to permanent visual impairment [12]. Although not reported often, these pathogens are likely very common in countries that lack clean water supply. Of the 884 million people worldwide who lack access to clean water, 40% are from Sub‐Saharan Africa, and more than 300 million of these have limited access to safe drinking water, with many also unable to access safe water for bathing, washing and recreation [13, 14, 15].

A large number (55%–85%) of rural households in Uganda access water that do not meet the minimum required standard [16, 17, 18]. Poor water quality in rural areas of Uganda could be due to factors such as high population pressure and increased industrialisation, consequently leading to untreated wastewater, sewage and other dangerous organic matter and chemicals entering the water supply system [19, 20]. Untreated organic matter [21] and that containing faecal coliform or parasites can be harmful to the environment and the end‐users [22]. When this happens, the end‐users are faced with a variety of health challenges. Periodic outbreaks of waterborne diseases were reported in Uganda within the period of 2009–2021 mainly due to poor water safety and hygiene‐associated causes [16, 20, 23–25]. Despite the periodic outbreaks, the government has done little to provide appropriate control measures. The most effective way to prevent these types of disease outbreaks is to spread information about the potential waterborne diseases and how to avoid them.

It is imperative to increase research on waterborne pathogens that could potentially be a threat to human and animal health in Uganda, as well as the dissemination of information about those pathogens. This study was designed to determine the presence of waterborne protozoan and helminth parasites in the Queen Elizabeth Protected Area (QEPA), an area which receives the largest number of domestic and international tourists in Uganda and has a rapidly growing local population .

## METHODS

### Study design, setting and sampling strategy

**FIGURE 1:**
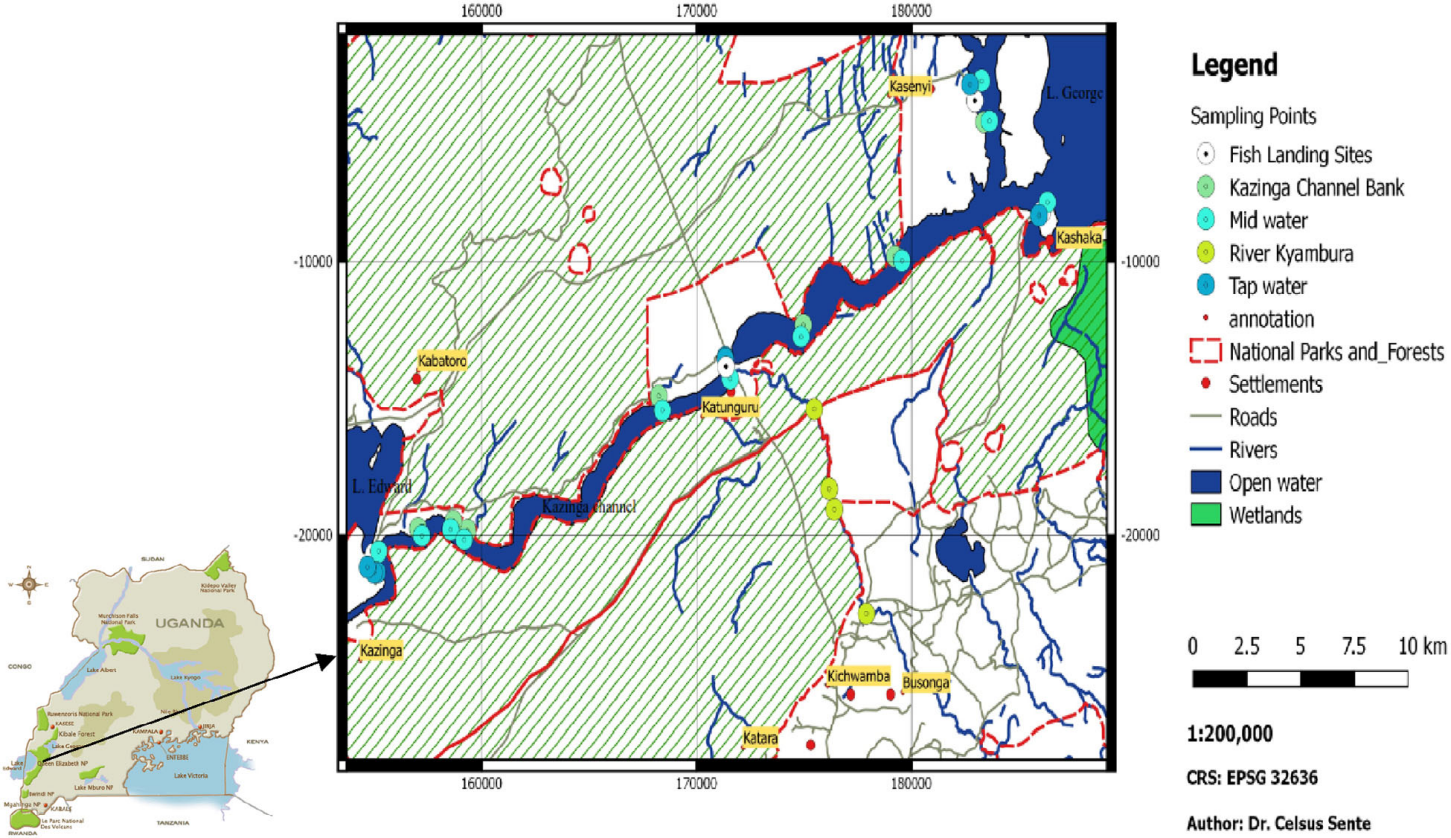
Study area showing sampling sites.

This was a cross‐sectional study conducted in 2014. The sampling locations were chosen purposively, considering their proximity to the end‐users. Naturally flowing (environmental) water samples were collected from areas where locals obtain water for domestic use as well as where they engage in recreational activities such as swimming (Figure 1). The water samples were collected from piped tap water (PTW) and natural water sources, namely river Kyambura (RK), Kazinga Channel banks (KCB), middle of Kazinga Channel (MKC) and fish landing sites (FLS). A total number of 324 natural surface water and 84 PTW samples were collected. The water samples were stored at room temperature and transported to the Makerere University Parasitology Laboratory immediately, within 48 h.

The research was carried out in Uganda's QEPA (Figure 1), located at a latitude of 0.2000 and a longitude of 30.0000. The protected area is characterised by Lakes George and Edward which are connected by the Kazinga Channel. QEPA is designated as a ‘Man and Biosphere Reserve’ by UNESCO and has 11 village enclaves, all of which have a rapidly expanding human population. The primary economic activities in these communities are fishing and livestock raising. Domestic water for human consumption in QEPA comes from natural sources such as the Kazinga Channel, RK, Lake George and Lake Edward. These natural water bodies are also used by the locals for swimming, bathing and washing clothes.

### Laboratory analysis

All the water samples were centrifuged at 1000 × *g* for 10 min to concentrate the oocysts, cysts and eggs [26]. A pellet was left at the bottom of the falcon tube after the supernatant was poured off. The pellet underwent direct wet smear, xenic cultivation, Ziehl–Neelsen's (ZN) staining, floatation and McMaster techniques to recover and quantify the organisms.

Direct wet mount was used mainly for protozoa trophozoites [27]. A small amount of concentrated water pellet was put on a microscope slide, and a drop of iodine was added. Observation was done under 10× and 40× objective.

For ‘xenic cultivation’, non‐nutritive media [28, 29] was seeded with 0.1 mL of a heat‐inactivated culture of *Escherichia coli* BL2 [29]. Pellets produced following centrifugation of water were carefully plated on NNA‐EI agar plates that had already been seeded. The plates were then placed in an incubator and set to 320°C for the night. The following day, each plate was placed in an individual polythene bag and then inverted and placed in an incubator at the same temperature for 7 days. For the detection of amoebae trophozoites, a Motic AE2000 Binocular inverted microscope (TED PELLA Inc.) was used. Counting the trophozoites was done using a haemocytometer [30].

A modified version of ZN's carbol‐fuchsin stain was used to detect the oocysts of *Cryptosporidium* spp. [31, 32]. After centrifugation, a few drops of water were placed on a slide and stained with ZN‐carbol‐fuchsin stain for 2 min. The slide was then rinsed with tap water to remove any remaining stains. After this step, the sample was rinsed with 3% hydrochloric acid in 70% ethanol and then with water from the tap. The finished object was then counterstained with Brilliant Green for 2 min before being rinsed with tap water to remove any excess stain. The slide was allowed to air‐dry before being examined under a microscope (10× and 40× objective).

Zinc sulphate floatation technique [29, 33] was used to identify protozoan cysts and helminth eggs. A solution of zinc sulphate with a specific gravity of 1.18–1.2 was placed inside of a test tube. A volume of 1.5 mL of the concentrated water was added to the ZnSO_4_ solution in the test tube, which was then stirred. Then, zinc sulphate solution was poured into the test tube until it was full. The full test tube was covered with a grease‐free slip and left for 15 min to give the cysts time to float. Then the cover slip was removed from the test tube and placed on the microscope slide to examine for the presence of protozoan cysts and helminth eggs (10× and 40× objective).

For the McMaster technique, the pellet was passed through a sieve into a dish containing 45 mL of saturated salt solution [33]. A sample of the mixture was placed in one of the McMaster chamber slides using a pipette and the procedure was repeated to fill the second chamber. The total number of helminth eggs and (oo)cysts in both the etched areas of the slide were counted and multiplied by 100 to determine the number of (oo)cysts/eggs per gram.

### Data analysis

Data was analysed using SPSS (IBM). Variables were summarised by the use of mean and standard error of the mean. The application of univariate analysis to compare prevalence across sampling sites was executed using cross‐tabulation with a *χ* ^2^ test. All variables with a *p*‐value of ≤0.05 were considered significant.

## RESULTS

### Overall prevalence of parasites

The overall percentage prevalence of the protozoan and helminth water parasites in the 408 water samples collected is presented in Table 1. *Cryptosporidium*, *Giardia*, FLA and helminths were identified.

**TABLE 1:** Overall prevalence of water parasites in Queen Elizabeth Protected Area (QEPA) in 2014 (*n* = 408).

Parasite	*N*	%
Protozoa		
Free living amoeba	231	56.6
*Cryptosporidium* spp.	156	38.2
*Giardia* spp.	149	36.5
*Eimeria* spp.	83	20.3
*Paramecium* spp.	11	2.7
Helminths		
Strongyles	156	38.2
*Ascaris* spp.	136	33.3
Strongyloides	42	10.5
*Toxocara* spp.	34	8.3
*Capillaria* spp.	13	3.2
Trematodes	101	25

### Prevalence and mean counts of parasites across different sampling sites in QEPA

Table 2 presents the percentage of parasites that were present at each water source (both natural and tap water) as well as the numerous natural sites that were taken into consideration. FLAs were the most prevalent parasite across all sources (PTW, 58.3%; natural water, 56.2%; R. Kyambura, 64.6%; KCB, 73.8%; FLS, 76.7% and KCM 32.6%). Meanwhile for helminth parasites, strongyle eggs were the most observed (PTW, 36.9%; natural water, 38.6%; R. Kyambura, 37.5% and FLS, 31.7%).

**TABLE 2:** Prevalence and mean counts of parasites across water sources in Queen Elizabeth Protected Area (QEPA) in 2014.

Parasite		Source					
Protozoa		PTW (*n* = 84)	Environ water (*n* = 324)	R. Kyambura (*n* = 48)	KCB (*n* = 84)	FLS (*n* = 60)	MKC (*n* = 132)
FLA	(+) (%)	49 (58.3)	182 (56.2)	31 (64.6)	62 (73.8)	46 (76.7)	43 (32.6)
	Mean (±SEM)	16.50 ± 2.67	14.25 ± 1.36	9.42 ± 2.72	16.00 ± 2.62	27.33 ± 5.64	7.53 ± 1.90
*Cryptosporidium* spp.	(+) (%)	22 (26.2)	134 (41.4)	14 (29.2)	62 (73.8)	28 (46.7)	30 (22.7)
	Mean (±SEM)	45.24 ± 9.34	92.16 ± 7.24	37.50 ± 9.24	223.81 ± 21.33	101.67 ± 16.03	53.79 ± 10.28
*Giardia* spp.	(+) (%)	32 (38.1)	117 (36.1)	12 (25)	38 (45.2)	32 (53.3)	35 (26.5)
	Mean (±SEM)	83.33 ± 14.15	101.72 ± 8.016	43.75 ± 11.87	152.38 ± 21.39	148.33 ± 22.61	81.06 ± 13.95
*Eimeria* spp.	(+) (%)	21 (25)	62 (19.1)	3 (6.3)	24 (28.6)	12 (20)	23 (17.4)
	Mean (±SEM)	51.19 ± 12.84	40.19 ± 4.87	12.50 ± 7.66	47.63 ± 9.94	46.67 ± 15.12	35.61 ± 7.98
*Paramecium* spp.	(+) (%)	0	11 (3.4)	3 (6.3)	6 (7.1)	0	2 (1.5)
	Mean (±SEM)	0	3.80 ± 1.31	8.33 ± 5.012	11.91 ± 5.46	0	1.14 ± .84
**Helminths**							
*Trematodes*	(+) (%)	16 (19)	85 (26.2)	8 (16.7)	30 (35.7)	12 (20)	17 (12.9)
	Mean (±SEM)	47.62 ± 11.67	49.51 ± 6.36	18.75 ± 6.42	114.29 ± 23.1	46.67 ± 13.12	28.79 ± 7.18
*Toxocara* spp.	(+) (%)	10 (11.9)	24 (7.4)	2 (4.1)	10 (11.9)	0	12 (9.1)
	Mean (±SEM)	34.52 ± 11.79	21.81 ± 4.04	4.17 ± 2.91	34.52 ± 11.67	0	21.97 ± 6.39
*Ascaris* spp.	(+) (%)	13 (15.5)	123 (38)	8 (16.7)	44 (52.4)	11 (18.3)	60 (45.5)
	Mean (±SEM)	35.71 ± 10.11	94.12 ± 7.95	16.67 ± 5.44	164.28 ± 22.57	53.33 ± 17.38	133.33 ± 14.85
Strongyles	(+) (%)	31 (36.9)	125 (38.6)	18 (37.5)	40 (47.6)	19 (31.7)	48 (36.4)
	Mean (±SEM)	152.38 ± 30.88	171.32 ± 23.66	83.33 ± 25.50	333.33 ± 99.42	111.67 ± 30.54	139.39 ± 23.65
Strongyloides	(+) (%)	8 (9.5)	34 (10.5)	0	16 (19)	8 (13.3)	10 (7.6)
	Mean (±SEM)	14.27 ± 5.12	14.71 ± 2.42	0	21.43 ± 5.10	21.67 ± 7.55	12.88 ± 4.74
*Capillaria* spp.	(+) (%)	0	13 (4)	0	0	6 (10)	7 (5.3)
	Mean (±SEM)	0	6.62 ± 2.02	0	0	16.67 ± 6.79	12.88 ± 5.31

Abbreviations: FLA, free‐living amoeba; FLS, fish landing site; KCB, Kazinga Channel bank; MKC, mid Kazinga Channel; PTW, piped tap water; SEM, standard error of the mean.

Natural water had significantly higher mean values compared to tap water (PTW) for *Giardia* spp., *Cryptosporidium* spp. and *Ascaris* spp. On the other hand, KCB and FLS compared to R. Kyambura and MKC had more parasite burden (Table 2).

## DISCUSSION

FLA and helminths may have a potential effect on public health, even though they are not often emphasised as biological contaminants (SuppMat) of domestic water in Uganda. This, combined with the lack of information of the Ugandan local community towards the correct way to safely use water, creates an opportunity for water to serve as a hub for the transmission of parasites among humans, animals and the environment. Exposure to waterborne diseases can be caused by a confluence of factors, some of which include ignorance [10, 34, 35], environmental contamination, climate change [9] and improper waste disposal [6, 18]. In Uganda, published information about waterborne parasites is scarce and only contains a few organisms that have occurred through major disease outbreaks. Although studies have been done on certain waterborne parasites, a lot remains unknown in many of Uganda's naturally occurring water systems, from which the largest percentage of rural communities obtain their water for domestic use. Monitoring of naturally occurring water from lakes, rivers, waterholes and other groundwater types is not given much attention in Uganda, as evidently demonstrated by the scanty published information on waterborne parasites.

The elevated incidence of FLA in QEPA may be attributed to the existence of organic material derived from decomposing foliage as well as animal and human excrement that accumulates along the shores of water bodies, hence leading to the contamination of water supplies. This finding aligns with prior studies that have revealed a higher prevalence of the FLA in areas characterised by elevated levels of organic matter contamination [36, 37, 38]. Additionally, the formation of biofilms inside piped water systems is also major contribution in the survival and transmission of FLA [39, 40, 41].

The present study reports an overall prevalence of *Cryptosporidium* spp. and *Giardia* spp. from water at 38.2% and 36.5%, respectively. Although there is limited published work on waterborne parasites in Uganda to compare these values with, some work has been done on faecal *Cryptosporidium* spp. and *Giardia* spp. in animals and humans [42, 43, 44]. Elsewhere, in Ethiopia, 102 (26%) and 31 (8.1%) *Giardia* spp. and *Cryptosporidium* spp. infections were reported out of the 384 children's stool samples examined [45]. The findings by researchers reveal that *Cryptosporidium* spp. and *Giardia* spp. have been more studied in faeces than directly from water samples, masking the high risks to individuals getting the infections from contaminated water that is often falsely presumed clean and free of these parasites. Over the last decade, *Cryptosporidium* spp. and *Giardia* spp. have emerged as major waterborne pathogens affecting the gastrointestinal tract of a wide range of vertebrates including humans, livestock and non‐human primates [46, 47]. *Cryptosporidium* spp. and *Giardia* spp. are known to cause human Cryptosporidiosis and Giardiasis, respectively. These diseases are associated with severe protozoan diarrhoea which often results in considerable morbidities.

Helminth eggs were found in all the tap and natural water sources. Helminth eggs can directly or indirectly have a significant health effect on humans, depending on the helminth lifecycle and the level of contamination of the water consumed, consequently causing gastrointestinal helminthiasis in children and adults. Although some helminth categories found in the present study may not cause danger through ingestion of water, their presence in domestic water sources in Uganda is an indication of poor water quality. The potential lethality of helminth infections is frequently underestimated, yet specific helminthic infections can result in severe repercussions for human health [48]. For example, the presence of ascarids and big strongyles in significant numbers inside the intestinal lumen might lead to the occurrence of intestinal obstruction [49], whereas Strongyloides is recognised for its ability to induce abnormalities in the gastrointestinal, pulmonary and dermatologic systems [50].

In natural water environment, pathogens have been isolated widely from many water sources such as rivers, lakes, streams, water holes, roadside gutters and reservoirs that are used as sources of water for most rural dwelling households [21, 51, 52]. Often inadequately treated domestic water (drinking water, bathing, cooking and recreational water) has an abundance of these pathogens. Although most of these pathogens are ubiquitous and naturally occurring in nature in normal concentrations, they are exacerbated by the addition of higher concentrations of (oo)cysts and eggs from agricultural run‐off, urban wastewater effluents [47] and for the case of QEPA, from continued unnecessary high human and animal faecal contamination. The QEPA local communities have poor personal hygiene habits, and few poorly built latrines, most of which are already filled up, compelling many to digging small holes in the ground and defecating outside, on open land. When there is a heavy downpour of rain, the faecal material is washed off into the RK, Kazinga Channel, Lake George, Lake Edward and other water bodies that provide domestic water supply to the communities, and the protected area premises. Upon using this water, exposure to a variety of protozoa and helminth is not uncommon.

Based on the results of this study, the following suggestions have been made: (1) More research should be done to identify the parasites by species; (2) access to better water sources should be paired with access to better sanitation and hygiene; (3) regular water quality assessments should be done to identify pathogen types and levels of concentration; and (4) the optimisation of water treatment methods should be done to get the best chemicals that can kill parasites.

## CONCLUSION

FLA, *Cryptosporidium*, *Giardia* and helminths were detected in both natural and domestic water samples collected in the QEPA. The high prevalence in both natural and domestic water sources can be ascribed to inadequate environmental sanitation and hygiene practices. The contaminated environmental water is the primary source of piped water in QEPA, posing a significant risk of infection for visitors and locals who use PTW without taking the necessary steps to treat it.

The research results were formally delivered to the relevant authorities, accompanied by recommendations to push for the implementation of enhanced PTW systems within Uganda's protected areas. This is necessary in order to guarantee the welfare of the native population as well as the ever‐increasing number of visitors who travel to these locations.

## AUTHOR CONTRIBUTIONS

*Conceptualisation; field data collection; data curation; methodology; writing—original draft and editing*: Celsus Sente. *Data analysis*: Howard Onyuth and Andrew Tamale. *Development of field study designs; writing‐review and editing*: Bob Mali, Benigna Gabriela Namara, Jacob Gizamba Mugoya and Andrew Rwot Omara. All authors read and approved the final version of the manuscript.

## CONFLICT OF INTEREST STATEMENT

There are no conflicts of interest.

## FUNDING INFORMATION

This study received no external funding. Internal resources were provided to aid data collection and analysis.

## ETHICS STATEMENT

This particular piece of work does not require ethics approval. This study's data was derived in part from my postgraduate thesis. This research was approved by the Uganda National Council for Science and Technology (UNCST). A permit to enter the national park was also acquired from the Uganda Wildlife Authority (UWA).

## Supporting information

Supporting Information

## Data Availability

The majority of the information created or analysed during this study is presented in this article. The associated authors will provide the remaining data upon reasonable request.
