# Supplementary material for: Waterborne parasites in Uganda: A survey in Queen Elizabeth Protected Area
Source: Public Health Chall. 2023 Dec 11;2(4):e142. doi: 10.1002/puh2.142 (PMC12039553; doi:10.1002/puh2.142)
Supplement: Supplementary file 1 — Supporting Information [file PUH2-2-e142-s001.docx]

**Water contaminant candidates list for Uganda**

| Microbial contaminant name | Information |
| --- | --- |
| Faecal coliforms | Facultative anaerobic, rod-shaped, gram-negative, non-sporulating bacterium, that originates in the intestines of warm-blooded animals. In Uganda, they have been found in natural and domestic water [1]. Rapid recharge of the springs and other water bodies after rainfall leads to increased microbiological contamination [2]. |
| Total coliforms | The total coliform counts in 90% of the samples collected from Kampala exceeded the WHO guideline for portable water [1]. |
| Faecal streptococcus | Predominant in areas with low sanitation [2]. |
| *Salmonella* species | Outbreaks due to the microbes (typhoid) have been predominant in many areas of the country, with more reports in Kampala [3, 4]. Many strains isolated from several water sources in Kampala [5]. |
| *Escherichia coli* | Humans harbouring *E. coli* bacteria that is ≈75% similar to the one from primates [6]. High levels found in Kampala water [7]. |
| Fresh water strains  (*Actinobacteria*,  *Alpha*-*Betaproteobacteria*, *Bacteroidetes*,  and *Spirochaeta*) | 65 strains from surface freshwater habitats [8]. |
| *Legionella* species | Commonly isolated and is associated with industrial water plant and household water heaters [9]. |
| Hepatitis | Hepatitis A & E associated with water, mainly faecal oral route transmission [10, 11]. |
| *Cryptosporidium* species | Faecal-oral route. It is a big problem in children, pregnant women, and immunocompromised individuals [12, 13]. |
| *Giardia lamblia* | Faecal-oral route. A big problem in children, pregnant women, and immunocompromised individuals [14]. |
| *Mycobacterium* species | Tuberculous and non-tuberculous can exist in soil and water [15, 16] |
| *Shigella* species | Faecal-oral routes and many strains exist [17]. |
| *Helicobacter* species | Unexpected variation in the prevalence of *H. pylori* infection in Uganda [18]. |
| *Vibrio cholerae* | Responsible for many death in many areas of Uganda [19, 20]. |
|  |  |

**References**

1. Haruna, R., F. Ejobi, and E.K. Kabagambe, *The quality of water from protected springs in Katwe and Kisenyi parishes, Kampala city, Uganda.* Afr Health Sci, 2005. **5**(1): p. 14-20.

2. Howard, G., et al., *Risk factors contributing to microbiological contamination of shallow groundwater in Kampala, Uganda.* Water Res, 2003. **37**(14): p. 3421-9.

3. Parker, A.H., et al., *An assessment of microbiological water quality of six water source categories in north-east Uganda.* J Water Health, 2010. **8**(3): p. 550-60.

4. WHO, *Emergencies preparedness, and response to Typhoid fever, Uganda*. 2015, WHO Regional Office for Africa: USA.

5. Afema, J.A., et al., *Potential Sources and Transmission of Salmonella and Antimicrobial Resistance in Kampala, Uganda.* PLoS One, 2016. **11**(3): p. e0152130.

6. Goldberg, T.L., et al., *Forest fragmentation as cause of bacterial transmission among nonhuman primates, humans, and livestock, Uganda.* Emerg Infect Dis, 2008. **14**(9): p. 1375-82.

7. Byamukama, D., et al., *Determination of Escherichia coli contamination with chromocult coliform agar showed a high level of discrimination efficiency for differing fecal pollution levels in tropical waters of Kampala, Uganda.* Appl Environ Microbiol, 2000. **66**(2): p. 864-8.

8. Hahn, M.W., et al., *The filtration-acclimatization method for isolation of an important fraction of the not readily cultivable bacteria.* J Microbiol Methods, 2004. **57**(3): p. 379-90.

9. Anacarso, I., et al., *Influence of Legionella pneumophila and other water bacteria on the survival and growth of Acanthamoeba polyphaga.* Arch Microbiol, 2010. **192**(10): p. 877-82.

10. Teshale, E.H., et al., *Hepatitis E epidemic in Uganda.* Emerging Infectious Diseases, 2010. **16**(1): p. 126-129.

11. CDC. *Health Information for Travelers to UgandaTraveler View*. 2016 [cited 2016 20th May]; Available from: <http://wwwnc.cdc.gov/travel/destinations/traveler/none/uganda>.

12. Desai, N.T., R. Sarkar, and G. Kang, *Cryptosporidiosis: An under-recognized public health problem.* Trop Parasitol, 2012. **2**(2): p. 91-8.

13. Salyer, S.J., et al., *Epidemiology and molecular relationships of Cryptosporidium spp. in people, primates, and livestock from Western Uganda.* PLoS Negl Trop Dis, 2012. **6**(4): p. e1597.

14. McElligott, J.T., et al., *Prevalence of intestinal protozoa in communities along the Lake Victoria region of Uganda.* Int J Infect Dis, 2013. **17**(8): p. e658-9.

15. Kankya, C., et al., *Isolation of non-tuberculous mycobacteria from pastoral ecosystems of Uganda: public health significance.* BMC Public Health, 2011. **11**: p. 320.

16. Eaton, T., et al., *Isolation and characteristics of Mycobacterium avium complex from water and soil samples in Uganda.* Tuber Lung Dis, 1995. **76**(6): p. 570-4.

17. Legros, D., et al., *Antibiotic sensitivity of endemic Shigella in Mbarara, Uganda.* East Afr Med J, 1998. **75**(3): p. 160-1.

18. Baingana, R.K., J.K. Enyaru, and L. Davidsson, *Helicobacter pylori infection in pregnant women in four districts of Uganda: role of geographic location, education and water sources.* BMC Public Health, 2014. **14**: p. 915.

19. Bwire, G., et al., *The burden of cholera in Uganda.* PLoS Negl Trop Dis, 2013. **7**(12): p. e2545.

20. Bwire, G., et al., *Cross-Border Cholera Outbreaks in Sub-Saharan Africa, the Mystery behind the Silent Illness: What Needs to Be Done?* PLoS One, 2016. **11**(6): p. e0156674.
